# Resveratrol attenuated hydrogen peroxide-induced myocardial apoptosis by autophagic flux

**DOI:** 10.3402/fnr.v60.30511

**Published:** 2016-05-20

**Authors:** Chih-Yang Huang, Wei-Jen Ting, Chih-Yang Huang, Jing-Yi Yang, Wan-Teng Lin

**Affiliations:** 1 Graduate Institute of Basic Medical Science, China Medical University, Taichung, Taiwan; 2 School of Chinese Medicine, China Medical University, Taichung, Taiwan; 3 Department of Health and Nutrition Biotechnology, Asia University, Taichung, Taiwan; 4 Translation Research Core, China Medical University Hospital, Taichung, Taiwan; 5 Department of Hospitality Management, College of Agriculture, Tunghai University, Taichung, Taiwan

**Keywords:** resveratrol, oxidative stress, apoptosis, autophagy

## Abstract

**Background:**

Resveratrol is a Sirt-1-specific activator, which also exerts cardioprotective effects that regulate redox signalling during oxidative stress and autophagy during cardiovascular disease (CVD).

**Objective:**

This study investigated the protective effects of resveratrol against hydrogen peroxide-induced damage in cardiomyocytes.

**Design:**

In this article, hydrogen peroxide-induced autophagy and apoptosis in H9c2 cardiomyoblasts were studied at an increasing concentration from 0 to 100 µM.

**Results:**

Resveratrol pretreatment with concentrations of 10, 20, and 50 µM inhibits autophagic apoptosis by increasing p-Akt and Bcl-2 protein levels in H9c2 cells. Interestingly, resveratrol treatment activates the Beclin-1, LC3, p62, and the lysosome-associated protein LAMP2a within 24 h of administration.

**Conclusions:**

These results suggest that resveratrol-regulated autophagy may play a role in degrading damaged organelles in H9c2 cells rather than causing apoptosis, and this may be a possible mechanism by which resveratrol protects the heart during CVD.

Oxidative stress caused by reactive oxygen species (ROS) is usually more than endogenous antioxidant defence mechanisms, which protect DNA, proteins, and carbohydrates against oxidation. Atherosclerosis, hypertension, diabetes heart failure, and the pathogenesis of cardiovascular disease (CVD) are related to oxidative stress (1). ROS, including hydrogen peroxide (H_2_O_2_) and peroxynitrite, are likely involved in the pathogenesis of myocardial ischemia-reperfusion injury (2). In 1966, Christian de Duve discovered that autophagy could eliminate damaged organelles, such as endoplasmic reticulum (ER) and mitochondria, through lysosome-dependent degradation (3). Simultaneously, autophagy provides energy that enhances survival under nutrient starvation by utilizing a dynamic recycling system (4).

Autophagy is triggered by inflammation, hypoxia, oxidized lipoprotein, ER stress, and ROS (5–7). When mammalian target of rapamycin (m-TOR) activity is inhibited by rapamycin, the autophagy activator ULK1 forms complexes (8). Next, LC3B-I and the phosphatidylethanolamine (PE) membrane combine to form LC3B-II (9, 10). Exogenous ROS can also induce autophagy, and ROS-generating drugs can promote autophagy. However, the lysosomal pathway of self-degradation has essential pro-survival functions (11). Autophagy in cardiomyocytes results in metabolic profit and loss (12). Furthermore, the regulation of autophagy occurs via metabolic and stress signalling pathways in the heart; therefore, autophagy has important functions in the myocardium, and its dysregulation is implicated in a wide variety of cardiovascular pathologies (13, 14).

Resveratrol, which is considered to be a cardioprotective compound, is a polyphenolic compound found in natural products (15). Recently, it was shown that resveratrol can prevent or treat cancer, heart disease, ischemic injuries, diabetes, pathological inflammation, and oxidative stress injuries (16–19). In this study, the cardiac myoblast H9c2 cell line was used to investigate the cardioprotective effects of resveratrol heart during H_2_O_2_-induced oxidative stress. The results show that the H_2_O_2_ treatment reduces Sirt-1 expression directly in H9c2 cells and causes H9c2 cells apoptosis. Resveratrol treatments attenuated H_2_O_2_-induced damage, enhanced Sirt-1 expression, and reduced autophagic flux in H9c2 cells.

## Methods and materials

### Cell culture

H9c2 cells were purchased from the Bioresource Collection and Research Center (BCRC, Taipei, Taiwan) and were cultured in Dulbecco's modified essential medium supplemented with 10% fortified bovine calf serum and incubated at 85% humidity in a 5% CO_2_ at 37°C. The H9c2 cells were starved in serum-free medium for 12 h and then pretreated with or without resveratrol for 2 h prior to the H_2_O_2_ treatments. The dorsomorphin (compound C) and the other inhibitors were used to pretreat H9c2 cells for 1 h before H_2_O_2_ stimulation.

### Mitochondrial emergence potential stain (JC-1)

H9c2 cells were pre-seeded at 2×10^4^ cells/well in 24-well plates for each test. After the indicated treatments, the media in the plates were removed, and the cells were washed with PBS. Next, JC-1 stain solution was added to each well at 37°C for 20 min, after which the JC-1 stain solution was removed, and the cells were washed with wash buffer. Fluorescence was visualized using a fluorescence microscope coupled with an image analysis system.

### Western blotting analysis

The protein concentration of each sample was determined using the Lowry protein assay. Protein samples for the western blotting assay were separated by 12% SDS polyacrylamide gel electrophoresis (SDS-PAGE) with a constant voltage of 75 V for 120 min. The proteins were then transferred to Hybond-C membranes (GE healthcare UK Ltd., Little Chalfont, Buckinghamshire, UK) at 50 V for 3 h. Hybond-C membranes with protein were incubated in 3% bovine serum albumin (BSA) in tricine buffer solution (TBS). Next, primary antibodies, including β-Actin (sc-47778, Santa Cruz Biotechnology, Dallas, TX, USA), Sirt-1 (sc-74465, Santa Cruz Biotechnology), p-Akt (sc-7985, Santa Cruz Biotechnology), m-TOR (#2983, Cell Signalling, Danvers, MA, USA), p-AMPK (AMP-activated protein kinase) (#2535, Cell Signalling), ULK-1 (#4773, Cell SignallingDanvers, MA,), p62 (#5114, Cell Signalling), Beclin-1 (#3738, Cell Signalling), Atg5 (#12994, Cell Signalling), LC3B I/II (#2775, Cell Signalling), LAMP2a (ab18528, Abcam, Cambridge, UK), cytochrome c (sc-13560, Santa Cruz Biotechnology), caspase 3 (sc-7148, Santa Cruz Biotechnology), Bax (sc-526, Santa Cruz Biotechnology), and Bcl-2 (sc-7382, Santa Cruz Biotechnology), were added to the membranes to recognize the proteins. Following primary antibody incubation, the membranes were incubated with horseradish peroxidase–linked secondary antibodies at room temperature for 1 h. Anti-rabbit, anti-mouse, or anti-goat IgG were used as the secondary antibodies, and the membranes were washed with TBS for 1 h. The blotting results were imaged with Fujifilm LAS-4000 (GE Healthcare UK Ltd.).

### Statistical analysis

All results were obtained from individual duplicates, and the experiments were performed at least three times. The results are presented as the group mean±standard deviation (SD). A one-way analysis of variance was used to determine the overall statistical significance for the means of the four experimental groups. A *p*-value less than 0.05 was considered to be significant. Statistical analyses were performed using SigmaPlot v.10.0 software.

## Results

### Hydrogen peroxide-induced autophagy and apoptosis in H9c2 cells

**Fig. 1:**
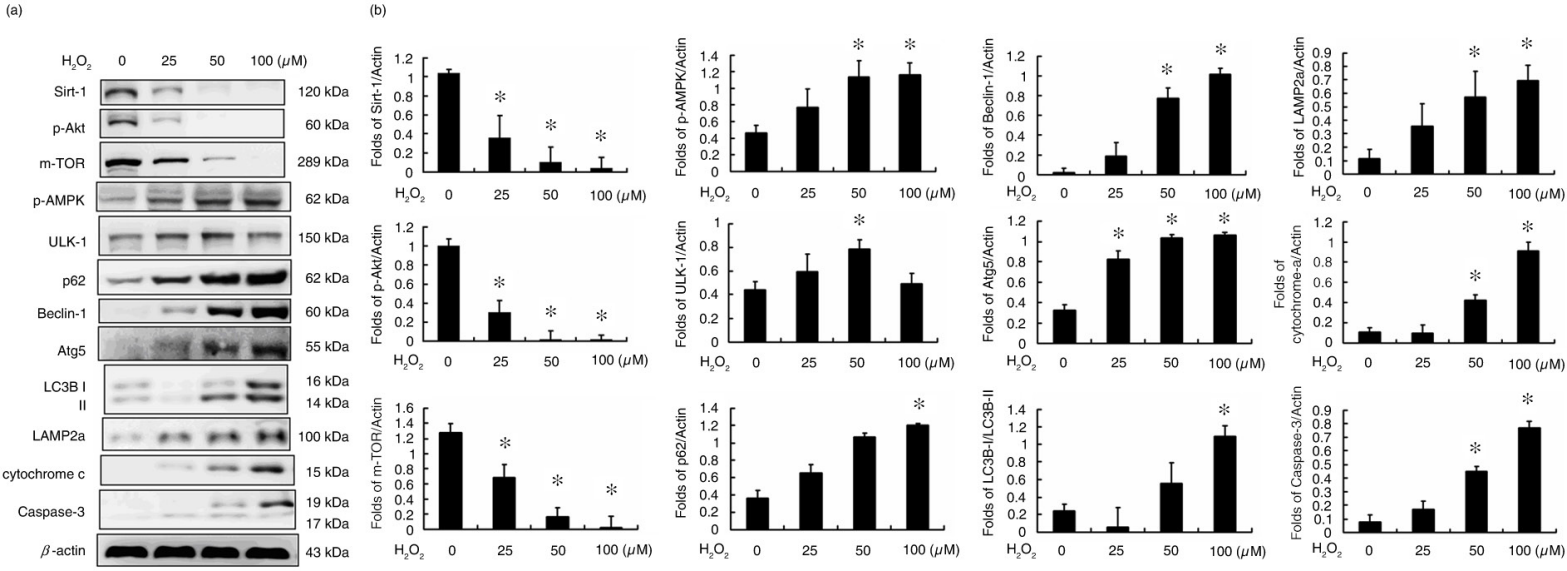
Hydrogen peroxide-induced autophagy and apoptosis in H9c2 cells. (a) The protein markers of survival, autophagy, and cell apoptosis pathways. (b) The fold change in the expression of each protein in hydrogen peroxide-treated H9c2 cells. (*P<0.01 compared with the control group).

In this study, the protein analysis determined that the expression levels of Sirt-1, p-Akt, and an m-TOR were dose dependently reduced in H_2_O_2_-treated H9c2 cells (Fig. 1). In addition, the levels of autophagy-related proteins, such as p-AMPK, unc-51, autophagy activating kinase 1 (ULK1), nucleoporin p62 (p62), Beclin-1, autophagy protein 5 (ATG5), LC3B, and LAMP2a, were also affected by H_2_O_2_ treatment of the H9c2 cells, suggesting that autophagy is induced by H_2_O_2_ treatment (Fig. 1). Further analysis indicated that caspase-3 expression was also increased by H_2_O_2_-treatments, which resulted in the release of cytochrome c (Fig. 1).

### Hydrogen peroxide-induced autophagy is regulated by the AMPK-ULK signalling pathway

**Fig. 2:**
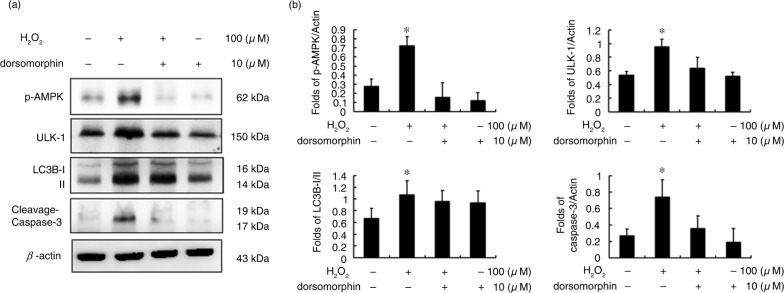
Hydrogen peroxide-induced autophagy is regulated by the AMPK-ULK signalling pathway. (a) Protein markers of the AMPK-ULK regulated pathway. (b) The fold change in expression for each protein in H9c2 cells with the indicated treatments. (*P<0.01 compared with the control group).

To identify the key pathway involved in H_2_O_2_-induced autophagy in H9c2 cells, dorsomorphin (compound C) was used to block AMPK activity. Indeed, p-AMPK protein levels were reduced after 10 µM dorsomorphin treatment (Fig. 2). The ULK-1 and LC3B proteins were downregulated after AMPK inhibition. Moreover, caspase-3 was also inhibited in dorsomorphin-treated H9c2 cells.

### H9c2 cardiomyocyte cell apoptosis via autophagy

**Fig. 3:**
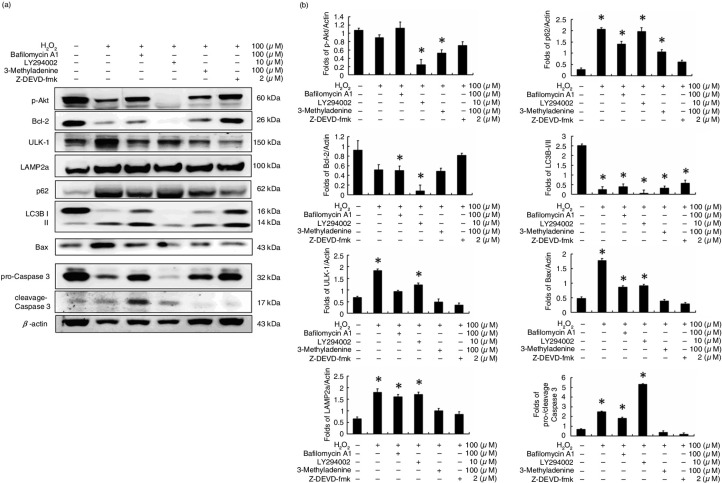
H9c2 cardiomyocyte cell apoptosis via autophagic pathway. (a) Protein markers of survival, autophagy, and cell apoptosis pathways. (b) The fold change in expression for each protein in hydrogen peroxide-treated H9c2 cells. (*P<0.01 compared with the control group).

Based on the results of Fig. 2, caspase-3 expression was reduced in a manner dependent on AMPK inhibition. This suggests a relationship between autophagy and cell apoptosis in H_2_O_2_-treated H9c2 cells. Here, bafilomycin A1 (100 µM) was used to co-treat H9c2 cells with H_2_O_2_. The results show that bafilomycin A1 inhibits degradation of the autophagosome and causes more significant cell apoptosis in H_2_O_2_ co-treated H9c2 cells (Fig. 3). Furthermore, a PI3K-Akt inhibitor (LY294002, 10 µM) was used to co-treat H9c2 cells with H_2_O_2_ (Fig. 3). The results showed not only autophagy but also cell apoptosis in H9c2 cells. However, when H9c2 cells were co-treated with a specific autophagy inhibitor (3-methyladenine, 100 µM) and H_2_O_2_, the levels of autophagy biomarker proteins, such as ULK-1, p62, and LC3B, were reduced. Furthermore, cell apoptosis was reduced for H9c2 cells co-treated with 3-methyladenine (100 µM) and H_2_O_2_, which was similar to the results of caspase-3 inhibitor (z-DEVD-fmk, 2 µM) and H_2_O_2_ co-treatment of the H9c2 cells (Fig. 3). These results suggest that increased autophagy will lead to cell apoptosis in H_2_O_2_-treated H9c2 cells.

### Hydrogen peroxide-induced mitochondrial membrane potential decrease and protection by resveratrol treatment in H9c2 cells

**Fig. 4:**
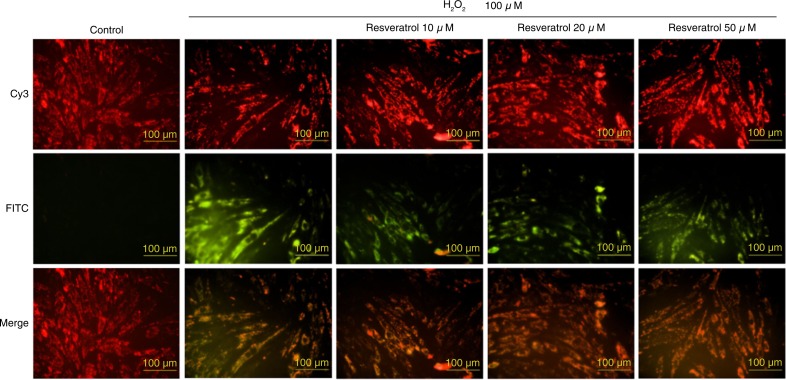
Hydrogen peroxide-induced mitochondrial membrane potential decrease and protection by resveratrol treatment in H9c2 cells.

The decrease in mitochondrial membrane potential can be visualized using a JC-1 staining assay. In the H_2_O_2_ treatment only group, red represents the normal mitochondria and green represents H9c2 cells with mitochondrial membrane potential instability (Fig. 4). After 4 h of H_2_O_2_ pretreatment, resveratrol (10, 20 and 50 µM) stabilized the mitochondrial membrane potential of the H9c2 cells.

### Resveratrol reduces hydrogen peroxide-induced autophagy and cell apoptosis in H9c2 cells

**Fig. 5:**
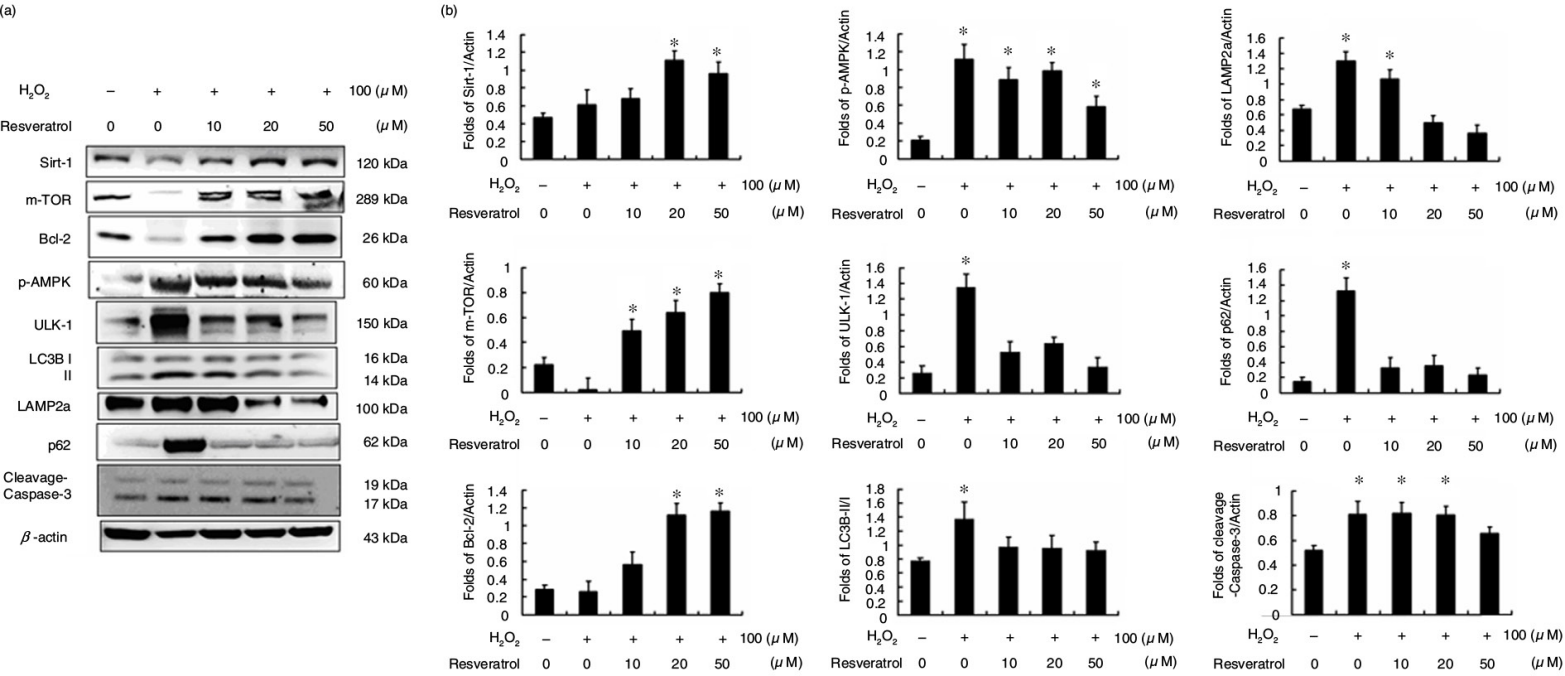
Resveratrol reduces hydrogen peroxide-induced autophagy and cell apoptosis in H9c2 cells. (a) Protein markers of survival, autophagy, and cell apoptosis pathways. (b) The fold change of expression for each protein in hydrogen peroxide-treated H9c2 cells. (*P<0.01 compared with the control group).

In this study, H9c2 cells were treated with or without H_2_O_2_ for 4 h and were then treated with or without resveratrol at different concentrations (0, 10, 20, and 50 µM). Protein analysis showed that autophagy and apoptosis were induced by H_2_O_2_ treatment only in H9c2 cells (Fig. 5). After resveratrol treatment, H_2_O_2_-induced autophagy and cell apoptosis were reduced in a dose-dependent manner.

## Discussion

Previous studies revealed that the H_2_O_2_ treatment induces cell apoptosis through caspase-3 activation and cytochrome c expression in H9c2 cells (20). Past evidence indicates that AMPK is an important physiological energy sensor that balances energy supply and demand, and regulates cellular processes (21). Treatment with H_2_O_2_ will cause a significant increase in the concentration of p-AMPK. Therefore, H_2_O_2_-activated, autophagy-related proteins may inhibit m-TOR activity through p-AMPK activation. The study also found that the apoptotic proteins cytochrome c and caspase-3 also play significant roles, indicating that H_2_O_2_-induced autophagy may affect the activity of apoptotic proteins (Fig. 1).

AMPK is activated when the ATP yield decreases intracellularly. AMPK plays key roles in regulating growth and is also involved in cell functions and processes associated with autophagy (22). Our previous studies showed that H_2_O_2_ leads to AMPK phosphorylation and inhibits m-TOR activity; therefore, we hypothesized that autophagy activation occurs through the AMPK pathway. Here, we used the AMPK inhibitor dorsomorphin (compound C) to determine whether H_2_O_2_ induced autophagy by activation of the AMPK pathway (Fig. 2). After treatment with dorsomorphin, H_2_O_2_-induced autophagy proteins, such as ULK1 and LC3B, were significantly decreased, and the expression of the pro-apoptotic protein caspase-3 was reduced after compound C treatment (Fig. 2). This demonstrates that H_2_O_2_-induced autophagy may also affect the activity of downstream apoptotic proteins.

It was recently found that autophagy exerts cell protective effects against doxorubicin-induced toxicity, hypoxia-reoxygenation, and ischemia-reperfusion injury (23, 24). However, the mechanisms underlying these effects remain complex. The cardioprotective effects of resveratrol may be mediated by Sirt-1 activation. In our previous study, Sirt-1 activation was highly associated with the PI3K-Akt pathway (25). In this study, we used the PI3K specific inhibitor LY294002 to block p-Akt expression in H_2_O_2_-treated H9c2 cells, which increased the apoptosis of these cells (Fig. 3). Moreover, the autophagy inhibitor 3-methyladenine decreased H_2_O_2_-induced cell apoptosis in H9c2 cells, similar to the caspase-3 specific inhibitor z-DEVD-fmk.

These results are consistent with those of a previous study that showed that resveratrol has multiple protective functions, particularly functions related to antioxidant activity *in vivo* (26). Further elucidation of the role of resveratrol in protecting cells and decreasing death will increase the effectiveness of its administration. H_2_O_2_ is extensively used as an inducer of oxidative stress *in vitro* (27). The JC-1 staining assay showed that H_2_O_2_ treatment of H9c2 cells resulted in H_2_O_2_-induced damage to the mitochondrial membrane potential and unstable green fluorescence (Fig. 4). Furthermore, resveratrol significantly protects H9c2 cells (Fig. 5).

In conclusion, our current findings show that resveratrol treatment may reduce H_2_O_2_-induced autophagy and cell apoptosis in H9c2 cells through Sirt-1 and p-Akt to regulate cardiac survival pathways. Our results suggest that resveratrol treatment may be beneficial for CVD.
